# Treatment of early uterine sarcomas: disentangling adjuvant modalities

**DOI:** 10.1186/1477-7819-7-38

**Published:** 2009-04-08

**Authors:** Flora Zagouri, Athanasios-Meletios Dimopoulos, Stelios Fotiou, Vassilios Kouloulias, Christos A Papadimitriou

**Affiliations:** 1 Department of Clinical Therapeutics, "Alexandra" Hospital, University of Athens, School of Medicine, Athens, Greece; 2 Aretaieion Hospital, University of Athens, School of Medicine, Athens, Greece; 3 Attikon Hospital, University of Athens, School of Medicine, Athens, Greece

## Abstract

Uterine sarcomas are a rare group of neoplasms with aggressive clinical course and poor prognosis. They are classified into four main histological subtypes in order of decreasing incidence: carcinosarcomas, leiomyosarcomas, endometrial stromal sarcomas and "other" sarcomas. The pathological subtype demands a tailored approach. Surgical resection is regarded as the mainstay of treatment. Total abdominal hysterectomy and bilateral salpingo-oophorectomy represents the standard treatment of uterine sarcomas. Pelvic and para-aortic lymph node dissection in carcinosarcomas is recommended, given their high incidence of lymph node metastases, and may have a role in endometrial stromal sarcomas. Adjuvant radiation therapy has historically been of little survival value, but it appears to improve local control and may delay recurrence. Regarding adjuvant chemotherapy, there is little evidence in the literature supporting its use except for carcinosarcomas. However, more trials are needed to address these issues, especially, their sequential application. Patients with uterine sarcomas should be referred to large academic centers for participation in clinical trials.

## Background

Uterine sarcomas are rare neoplasms comprising 1% of all gynaecologic malignancies and 4–9% of all malignant uterine neoplasms [1,2]. It is estimated that the incidence of uterine sarcomas varies between 0.5 and 3.3 cases per 100.000 females per year [3]. Uterine sarcomas usually display aggressive clinical behaviour, with a great tendency to local recurrence and even greater to distant spread [4-7]. Due to their low incidence and the fact that they lack a preinvasive stage, there is no established practice for screening these tumours. The rarity of uterine sarcomas and their often aggressive clinical course has resulted in a relatively limited amount of literature.

The staging of uterine sarcomas is based on the International Federation of Gynecology and Obstetrics staging system for uterine corpus cancer. According to WHO classification, uterine sarcomas are classified into four main histological subtypes in order of decreasing incidence: carcinocarcomas, leiomyosarcomas, endometrial stromal sarcomas and "other" sarcomas [8]. Unfortunately, clinical-trial reports and literature reviews often include a broad range of histological subtypes of sarcoma, which restricts interpretation and application of results. Response rates from protocols with multiple subtypes should consequently be interpreted with caution. Therefore, the effort to tailor the approach to patients with uterine sarcomas by pathological subtype seems mandatory.

## Carcinosarcomas (CSs)

### Epidemiology and Tumour Features

Carcinosarcomas represent a highly aggressive subtype of uterine malignancy [9-12]. CSs comprise both malignant epithelial and malignant sarcomatous components [13-15]. The differentiation of the epithelial component is usually glandular with the endometrioid adenocarcinoma being the most common epithelial type. The mesenchymal components may be "homologous", similar to tissues normally present in the uterus, or "heterologous," resembling tissue foreign to the uterus [12-15]. Patients with CSs usually present with a bulky polypoid mass extending into and even through the endocervical canal [16,17].

The recurrence rate for stage I and II CSs is approximately 50% [12,18]. Unfortunately, patients who present with early-stage disease confined to the uterus have a 2- to 5-year overall survival of approximately 50% (reviewed by Kanjeekal et al [19]). Factors associated with poor prognosis include stage, high grade of tumour, adnexal spread and lymph node metastasis. The prognostic relevance of histopathological variables, the grade of sarcomatous and epithelial component, the mitotic count of sarcomatous component, as well as the depth of myometrial invasion, the lymph-vascular space involvement and peritoneal cytology have been implicated in some studies as significant pathologic predictors [10,11,20-23]. It should, however, be noted that these findings did not emerge in the majority of studies conducted. Notwithstanding, the limited number of patients and the fact that most studies are not stratified for stage do not allow definitive conclusions to be drawn.

### Surgical Treatment

Surgical treatment of CSs should comprise exploratory laparotomy, total abdominal hysterectomy (TAH), bilateral salpingo-oophorectomy (BSO), omentectomy, aspiration of abdominal fluid for cytologic evaluation, pelvic and para-aortic lymph node dissection and tumor debulking at the time of presentation [12,13,16,17,19,24]. Lymph node dissection is indicated for CSs given their high incidence of lymph node metastases [18,25,26]. Moreover, the number of lymph nodes collected in stage I-II patients, who underwent pelvic and para-aortic lymph node sampling, has been correlated with the risk of recurrence and survival [25,26]. Surgical debulking in patients with uterine CSs should be attempted, given that patients with minimal residual disease may have a longer survival than those left with gross residual disease after surgical debulking [12]. More accurate surgical staging of CSs, as with endometrial cancer, may allow physicians to better assess the need for adjuvant treatment.

### Radiation Therapy

Historically, treatment for uterine CSs has included adjuvant pelvic radiation therapy with or without brachytherapy [12]. Until 2007, no well-controlled, randomized studies had been published, and most reports were based on small non-randomized trials [26-28]. The best conclusion that could be drawn from these reports was that the routine place of adjuvant pelvic radiation was limited as it only led to a statistically significant reduction of recurrences within the radiation field, but did not confer to overall survival [26-29]. Nonetheless, adjuvant radiotherapy has been shown to improve survival for patients with early-stage CSs who had not undergone lymph node dissection [29,30].

In their retrospective analysis, Callister et al [31] found that patients treated with pelvic irradiation had a lower rate of pelvic recurrence than those treated with surgery alone (28% versus 48%, p = 0.0002), but the 5-year overall survival and distant metastasis rates were not significantly different. Interestingly, patients treated with pelvic irradiation had a longer median time to any distant relapse (17.3 versus. 7.0 months, p = 0.001) than those treated with surgery alone. In another retrospective analysis of 2461 women with uterine CSs within the SEER (Surveillance, Epidemiology, and End results program) database of the US National Cancer Institute, radiation therapy predicted an improved overall and disease specific survival [32]. Five-year overall survival rates were 41.5% and 33.2% (p < 0.001) for women receiving or not irradiation, respectively. More analytically, women with stage I-III disease experienced a benefit in overall survival (hazard ratio = 0.87, p = 0.03), while those with stage IV disease experienced benefit in both overall (HR = 0.63, p < 0.001) and uterine-specific survival (HR = 0.63, p = 0.004).

In 2007, GOG published the results of a randomized phase III study which compared patient outcome following treatment with adjuvant whole abdominal irradiation (WAI) versus chemotherapy with three cycles of cisplatin and ifosfamide (CIM) for patients with CS. Eligible patients had stage I-IV disease, no more than 1 cm postsurgical residuum and/or no extra-abdominal spread. FIGO stage was I+II in 44% of the enrolled, eligible women. Adjusting for stage and age, the recurrence rate was 21% lower for CIM patients than for WAI patients (relative hazard = 0.789, p = 0.245). The estimated death rate was 29% lower among the CIM group (relative hazard = 0.712, p = 0.085). Although the observed differences were not statistically significant, the authors seems to favor the use of combination chemotherapy in future trials [33].

In the mid-1980s, the European Organisation for Research and Treatment of Cancer (EORTC) Gynaecological Cancer Group proposed a trial to evaluate adjuvant radiotherapy in stage I+II uterine sarcomas (protocol 55874). The study opened in 1987 taking 13 years to accrue 224 patients and its results were first published in 2008. Patients were required to have undergone as a minimum, TAH and BSO and washings, but nodal sampling was optional. There were 103 LMSs, 91 CSs and 28 ESSs. Patients were randomised to either observation or pelvic radiation, 51 Gy in 28 fractions over 5 weeks. Analysis for all patients revealed a reduction in local relapse (p = 0.004), but no effect on either OS or PFS. Furthermore, no difference in either overall or disease-free survival was demonstrated among CS patients but there was an increased local control for those receiving radiation, while this benefit was not observed for women with LMSs. Prognostic factor analysis showed that stage, age and histological subtype were important predictors of behaviour [34].

In conclusion, CSs appears to behave like poorly differentiated endometrial carcinomas. Offering adjuvant pelvic radiation applies only to patients that have not undergone a complete surgical staging including lymph node staging. However, further randomized trials are necessary in order to clarify the exact role of adjuvant radiotherapy, especially in combination with platinum-based chemotherapy.

### Chemotherapy

The role of adjuvant chemotherapy in CSs is still under debate. In a prospective phase II GOG study, patients with completely resected stage I or II CSs received three cycles of adjuvant chemotherapy with ifosfamide and cisplatin. PFS and OS, respectively, were 54% and 52% at 84 months. The authors concluded that the impact of adjuvant ifosfamide and cisplatin on survival outcomes was unclear, while pelvic relapses remained a concern [35]. In a recently published retrospective analysis, 49 women with completely resected stage I-IV CSs received in the adjuvant setting either platinum-based chemotherapy with or without radiation therapy (pelvic or WAI), or radiation therapy alone. Three-year PFS for chemotherapy group (with or without radiation therapy) was 35% versus 9% for radiation therapy alone (HR = 1.74, p = 0.164), while the corresponding 3-year OS rates were 66% and 34%, respectively (HR = 2.02, p = 0.146). Interestingly, the majority of patients in the chemotherapy group were treated with paclitaxel and carboplatin [36]. When compared with other regimens, the latter combination seems also to improve the overall survival rate of patients with ovarian CSs [37].

### Endocrine therapy

In contrast to ESS, endocrine therapy has no place in the adjuvant treatment of CS. However, given that apparently 30% of these tumours express estrogen and/or progesterone receptors, hormone therapy may represent an option in chemotherapy pretreated patients with hormone receptor positive tumours [12].

## Leiomyosarcomas (LMSs)

### Epidemiology and Tumour Features

Smooth muscle cell tumours may be divided into three groups: benign (leiomyoma), malignant (leiomyosarcoma), and tumours of unknown malignant potential [38]. Uterine LMS is a rare tumour, accounting for approximately 1% of all uterine malignancies, and represents the second more common uterine sarcoma. According to an analysis of SEER program data, CS were the most common uterine sarcoma followed by LMS [3,12,39]. The incidence of LMS in patients operated for presumed leiomyoma is approximately 0.1–0.3% [40]. Unfortunately, due to the stromal rather than endometrial origin of the tumour, LMS is not often diagnosed preoperatively. Although some patients complain of pain or bleeding and undergo endometrial biopsy as a result of these symptoms, most women with LMS lack symptoms, while others present with a rapidly enlarging pelvic mass and thus do not undergo biopsy before surgery [12,16,17].

Sixty percent of women with LMS present with disease clinically limited to the uterus. Cure rates for these patients range from 20% to 60%, with rates depending on the success of primary resection [12,13]. The recurrence rate is approximately 70% for stage I and II disease, and the site of recurrence is often distant [12,18]. Favourable prognostic features to emerge in some studies include premenopausal status, low mitotic count, pushing margins, hyalinization, absence of necrosis, origin in a uterine leiomyoma, and tumour stage. However other studies question these findings [18,41-43]. The main criteria used to determine treatment and prognosis are: necrosis, nuclear atypia, and mitotic count [12,41,43].

### Surgical Treatment

Management practice varies for uterine LMS, and is poorly supported by evidence in the current literature, but total abdominal hysterectomy and bilateral salpingo-oophorectomy is considered to be the standard surgical treatment [4,12,13,16,44]. Contrary to CS, pelvic and/or para-aortic lymphadenectomy is not indicated for LMS, unless macroscopic extra-uterine disease is present; due to the low rates of negative nodes and the fact that the dominant pattern of recurrence in LMS is outside the pelvis and the abdominal cavity [13,16,17,44].

Ovarian preservation may be considered in premenopausal patients with early-stage leiomyosarcoma of the uterus as case-control investigations suggest that it does not adversely affect survival [13,44]. In a retrospective review of 208 patients with LMS of the uterine, the multivariate analysis showed that oophorectomy was associated with a significantly worse disease-specific survival [44].

### Radiation therapy

Pelvic radiation therapy has been used for adjuvant treatment of uterine LMS. However, although radiation therapy has been shown to reduce the pelvic relapse rate by 50%, studies have not demonstrated a significant survival benefit with this approach [12,27,31,44-46]. The results of the protocol 55874 coincide with this observation. This significant randomized trial demonstrated that patients with LMS did not show the same benefit from radiation as patients with CSs [34].

In patients with LMS, in contrast to patients with other uterine sarcomas, the dominant pattern of recurrence is outside the pelvis and abdominal cavity [47-49]. At least two thirds of patients with uterine LMS have some component of distant disease at first recurrence. Thus, although the rate of recurrence in the pelvis is not insubstantial, little is potentially gained by delivering pelvic radiation as a postoperative adjuvant treatment.

### Chemotherapy

No adjuvant treatment has been shown to improve survival, although prospective data are very limited. However, in a small phase II study, four cycles of adjuvant chemotherapy with gemcitabine and docetaxel in patients with completely resected stage I-IV, high-grade uterine LMSs resulted in 2-year PFS rates that appear superior to historical rates (this issue will be discussed more detailed in the final section of the article) [50].

### Endocrine therapy

Endocrine therapy has no place in the adjuvant treatment of patients with LMS.

## Endometrial Stromal Sarcoma (ESSs)

### Epidemiology and Tumour Features

ESS is a rare entity of uterine malignancy, accounting for 0.2–1% of all uterine malignancies and 6–20% of all uterine sarcomas [51-53]. Due to its rarity, ESS has only been reported in a few studies, and these were limited to case reports or retrospective analysis based on a small number of patients. These tumours are most commonly seen in premenopausal women, but age at presentation may range from 20 to 80 years [12]. Patients typically present with bleeding and pain [17,54]. Due to the fact that most patients with ESS have obvious symptoms, they are usually diagnosed at an early stage [54-56].

According to its pathological features ESS has been classified as low grade or high grade [12,55]. Low-grade ESS is characterized by fewer than 10 mitoses per 10 high-power fields and a lack of significant atypia and often expresses estrogen and progesterone receptors. It should be noted that nowadays, rather than including high-grade ESSs in the category of ESSs, they are grouped together with high-grade or undifferentiated uterine sarcomas (UUSs). This is important because the low-grade and high-grade variants have vastly different prognostic factors and therapeutic options [54-56].

ESS may arise from uterine stroma, adenomyosis, or possibly endometriosis [12,57]. They usually have an indolent clinical course with 5-year survival rates of 80% to almost 100%, but about 37–60% of patients eventually recur after a very long time and 15–25% die of the disease [13]. In ESS recurrences are usually local, but late recurrences may involve the lung and abdomen. Stage at presentation is the best predictor of recurrence risk [12].

ESSs frequently show positive immunoreactivity for ER and PR, which helps in differential diagnosis [57-68]. Reich et al [63], analysed ER and PR expression in a retrospective series of 21 patients with ESS. ER and PR were measured with monoclonal antibodies and the peroxidase-antiperoxidase method and a score was calculated, as for breast carcinoma, based on both the percentage of positive tumour cell nuclei and the staining intensity. The authors concluded that ER and PR expression in ESSs is heterogeneous. This finding may have implications for hormone therapy in the management of these tumours and may suggest that ER and PR should be routinely quantified in ESSs by immunohistochemical methods. Chu et al [62] evaluated the expression of ER-alpha and ER-beta in low grade ESS and they suggested that loss of ERβ expression may be a marker for malignancy. Accordingly, Balleine et al [59] showed that PR isoform expression, similarly to that of a normal endometrial stroma, is consistent with the highly differentiated phenotype of this tumor and that variant differentiation or disease recurrence was accompanied by an altered PR isoform profile that could impact on hormone response.

### Surgical Treatment

Surgical treatment of ESS typically includes an exploratory laparotomy, total abdominal hysterectomy and bilateral salpingo-oophorectomy, omental biopsy, and aspiration of abdominal fluid for cytologic evaluation [7,12,13,54,69,70]. If the tumour is palpable in the parametrium, a more extensive procedure, such as a radical hysterectomy, should be performed [12]. Lymph node sampling is not the standard of care, as nodal involvement by low-grade ESS is supposed to be rare [12]. In two recently published series nodal involvement was found in 33–45% of cases during primary or secondary surgical treatment, and lymphadenectomy has been suggested by the authors as part of the surgical treatment [71,72]. However, this finding was observed in patients with more advanced disease and, therefore, the role of lymphadenectomy in early stages still remains controversial. Ovarian ablation is necessary, as part of primary treatment, because estrogens may act as a trophic agent for these tumours [73].

### Radiation therapy

Adjuvant radiation therapy is effective treatment for patients with ESS due to excellent local control in all stages and good disease-specific survival in early stages [69,70]. Adjuvant radiation therapy clearly reduces the incidence of pelvic recurrence; however, in the majority of the studies conducted it has no effect on OS [70,74-77].

### Chemotherapy

See the final section of this review.

### Endocrine therapy

Low-grade endometrial stromal sarcomas are estrogen and progesterone receptor positive tumors [63,78]. In the past, hormonal therapy consisted of progestins for advanced, recurrent or metastatic low-grade ESS. Medroxyprogesterone acetate (MPA) and megestrole acetate are synthetic derivatives of progesterone that exert their activity after binding to the progesterone receptor [79]. Aromatase inhibitors [80-83] and GnRH analogues [84-86] have become new effective alternatives for first and second line treatment. The high recurrence rates after short disease free intervals in low-grade ESS patients were partly due to inadvertent growth stimulation during estrogen-containing hormone replacement therapy and tamoxifen treatment, which are contraindicated [73,78,87]. Recently, hormonal therapy has been introduced for the prevention of recurrences. However, it should be noted that well controlled randomised studies on ESSs have not been conducted and the majority of the results reported are based on small series.

## Undifferentiated Uterine Sarcomas (UUSs)

### Epidemiology and Tumour Features

Undifferentiated uterine sarcomas are high-grade epithelioid or spindle cell sarcomas that cannot be classified into one of the standard categories [4,13,35]. These tumours usually present with abdominal or pelvic pain in postmenopausal women and represent less than 5% of all uterine sarcomas [12]. They are characterized by an aggressive clinical behaviour, with a tendency to frequent and early recurrences and with 5-year survival rates of 25–55% [7,13,52]. Necrosis is a common finding [12,52]. The stage at diagnosis as well as the mitotic index have emerged in some studies as the most significant predictor of prognosis [12,52], a finding that has not been reported in other series [88].

### Surgical Treatment

The treatment of choice for undifferentiated uterine sarcomas is total abdominal hysterectomy and bilateral salpingo-oophorectomy [12,13,17,19,24]. There are no data in the literature to support or challenge lymph node dissection. While Gaducci et al [13] suggest that this surgical procedure is not advisable, Ramondetta et al [12] recommend lymph node dissection in order to determine the risk of recurrence.

### Radiation therapy

Radiation therapy is typically recommended for stage I and II UUSs [12,13,30]. However, concern regarding distant recurrences has led to the consideration of combining irradiation with chemotherapy.

### Endocrine therapy

Hormonal therapy plays no role for UUSs due to the lack of steroid receptor expression.

## Overview of adjuvant chemotherapy in early stage uterine sarcomas

### Older non-platinum containing regimens

The high incidence of distal metastasis in uterine sarcomas makes adjuvant chemotherapy an appealing option. Although adjuvant chemotherapy has been studied in a number of trials, considerable controversy still surrounds its use. The question is whether or not adjuvant chemotherapy can achieve a significant increase in disease-free and overall survival without major treatment-related toxicity. It should be noted, that due to the rarity of uterine sarcomas, very few prospective randomized studies have been conducted. Also, the non randomised clinical-trial reports often include a broad range of histological subtypes, which restricts interpretation and application of results.

The role of adjuvant chemotherapy and its type still remains unclear even for adult soft tissue sarcomas (STSs). The EORTC randomised patients with completely resected STSs to receive either the CyVADIC regimen (cyclophosphamide, vincristine, doxorubicin and dacarbazine) adjuvantly or no additional treatment [89]. With a median follow-up duration of 80 months, the 7-year relapse-free survival was higher and the local recurrence rate lower in the chemotherapy arm. Overall survival and the rates of distant metastases were not affected by chemotherapy. In contrast, in another randomized trial, Frustaci et al [90] demonstrated that 5 cycles of adjuvant chemotherapy with epirubicin and ifosfamide) significantly improved median disease-free (48 months versus 16 months, p = 0.04) and overall survival (75 months versus 46 months, p = 0.03) in patients with high-risk extremity STSs. An update of the study showed the 5-year overall survival estimate to be 66% and 46% (p = 0.04) for the chemotherapy and control arm, respectively [91]. The Cochrane Review performed a meta-analysis of 14 trials of adjuvant chemotherapy, involving 1568 patients with STSs. According to this meta-analysis chemotherapy improved the local recurrence-free interval (HR = 0.73), distant recurrence-free interval (HR = 0.70), and overall recurrence-free interval (HR = 0.75). The doxorubicin-based therapy yielded an advantage in terms of overall survival, but failed to achieve statistical significance (HR = 0.89) [92].

Although uterine sarcomas are different from STSs of the trunk and extremities, in terms of pathogenesis and biological behavior, and results of adjuvant chemotherapy in early stage STSs cannot be extrapolated in uterine sarcomas, some studies used either the VAC or the CyVADIC combination in the adjuvant setting. More specifically, in a retrospective analysis of stage I and II uterine sarcomas, Buschbaum et al [93] reported recurrent disease in 37% of patients who received adjuvant chemotherapy with the vincristine, dactinomycin and cyclophosphamide (VAC) regimen. In another study, Hannigan et al [94] retrospectively analyzed the outcome of patients who received after hysterectomy and radiotherapy VAC regimen or the combination of vincristine, doxorubicin and cyclophosphamide and concluded that neither the probability of survival nor the disease-free interval was improved by the addition of adjuvant chemotherapy. Adjuvant VAC was also utilized in a prospective, pilot study in patients with stage I uterine sarcomas. The authors reported that recurrence rate was significantly (p < 0.02) less than that for patients treated by surgery alone or by surgery plus radiation [95].

The impact of adjuvant CyVADIC on survival outcomes of patients with stage I uterine sarcomas was examined in two small prospective studies. Hempling et al [96], treated twenty women who underwent total abdominal hysterectomy and bilateral salpingo-oophorectomy with this multiagent chemotherapy regimen, but failed to show a significant impact on long-term survival in this group of patients. Progression-free survival for the entire population at 2 years was 80% and at 5 years 65%, while recurrence rates for pure sarcomas and carcinosarcomas did not differ significantly. In a more recently published study, Odunsi et al [97] reported results on adjuvant CyVADIC in 24 patients with a very long duration of follow-up. The estimated survival rate for the whole group was 88, 75, and 69% at 2, 5, and 15 years, respectively. Factors that did not affect survival included age, histology, and tumor grade. Based on their data, the authors suggested that CyVADIC may have a potential role in the adjuvant treatment of early-stage uterine sarcoma.

### Ifosfamide-containing regimens

Ifosfamide-based chemotherapy has been investigated in the adjuvant setting in three prospective, non-randomized trials. Kushner et al [98], treated patients with completely resected moderate-to-high grade uterine sarcomas with three cycles of adjuvant ifosfamide (1.5 g/m^2^/day × 3 consecutive days, repeated every 4 weeks). Median progression-free survival was 26 months with a corresponding 2-year rate of 60%. The 2-year overall survival rate was 100%, dropping to 64% at three years. In another French study conducted by Pautier et al [99], patients received three cycles of adjuvant chemotherapy with doxorubicin, cisplatin and ifosfamide (API) followed by radiotherapy. When compared to a group of patients treated with adjuvant radiotherapy alone, the 3-year disease-free survival was 43% in the radiotherapy group and 76% in the combined modality group. Of note, the two previously mentioned studies included a mixture of different histological types. On the contrary, only patients with CS were included in a prospective phase II GOG study, which utilized the combination of ifosfamide and cisplatin for stage I+II disease. Ifosfamide was administered 1.5 g/m^2^, intravenously over 1 h, and cisplatin was given 20 mg/m^2 ^followed by ifosfamide 1.5 g/m^2^/24 h as a continuous infusion (daily × 5, every 21 days, × 3 cycles). PFS and OS, respectively, were 69% and 82% at 24 months, and 54% and 52% at 84 months. Leukopenia was the most commonly reported, but manageable, toxicity. The authors concluded that the impact of adjuvant ifosfamide and cisplatin on survival outcomes was unclear, while pelvic relapses remained a concern [35]. In contrast, in their retrospective analysis of 31 patients with stage I or II uterine sarcomas of various histologies, Papadimitriou et al [4] found that adjuvant ifosfamide-containing chemotherapy resulted in statistically longer OS when compared with non-ifosfamide-containing chemotherapy.

The combination of ifosfamide (with MESNA uroprotection) and cisplatin (CIM regimen) was also used in a randomized phase III GOG study that enrolled 232 patients. This trial was designed to compare patient outcome following adjuvant treatment with WAI versus chemotherapy with three cycles of CIM in women with completely resected CSs. Adjusting for stage and age, the recurrence rate was 21% lower for CIM patients than for WAI patients (relative hazard = 0.789, p = 0.245). The estimated death rate was 29% lower among the CIM group (relative hazard = 0.712, p = 0.085) [33].

### Anthracycline-based chemotherapy

The role of adjuvant doxorubicin has been examined in a randomized GOG trial. After hysterectomy, 156 evaluable patients with stage I or stage II uterine sarcomas were randomly assigned to chemotherapy with doxorubicin for six months or to no further treatment. Pelvic irradiation (external or intracavitary) was optional before randomization. Of 75 patients receiving doxorubicin, 31 have suffered recurrences compared with 43 of 81 receiving no adjuvant chemotherapy, but this difference was not statistically significant. Moreover, there was no difference in progression-free interval or survival. The optional radiotherapy did not influence the outcome although there was a suggestion that vaginal recurrence was decreased by pelvic radiotherapy. The recurrence rates in specific histologies were not significantly different [100].

Anthracycline-based combinations have been used in CSs. In a phase II trial, 42 consecutive patients were treated with a combination containing etoposide 100 mg/m^2^, on days 1 and 2, cisplatin 50 mg/m^2 ^on day 1, and doxorubicin 50 mg/m^2 ^on day 1, repeated every 28 days. There were 23 patients with early-stage disease and 19 patients with advanced or recurrent disease. Among patients with stages I and II there were 5 (22%) recurrences and the 2-year overall survival rate was 92% [101]. Additionally, a sequential approach with adjuvant radiation therapy and chemotherapy has been correlated with improved clinical outcome. In a pilot study, conducted by Manolitsas et al [102], patients with stage I or II uterine CSs received after surgery tailored radiation therapy and chemotherapy, consisting of cisplatin and epirubicin. The survival rate for those patients who completed treatment according to the multimodality protocol was 95%, with a disease-free survival rate of 90%.

### Newer agents and combinations

The combination of gemcitabine and docetaxel has shown promising results as adjuvant chemotherapy for completely resected stages I-IV high grade uterine LMSs. As previously mentioned, Hensley et al [50] treated 25 patients with gemcitabine 900 mg/m^2^, on days 1 and 8, plus docetaxel 75 mg/m^2^, on day 8, every 3 weeks for 4 cycles. With a median follow-up of 49 months for all patients, 10 (45%) remained progression-free at 2 years, with a median progression-free survival of 13 months. Among the 18 patients with stages I or II disease, 59% remained progression-free at 2 years, with a median progression-free survival of 39 months. Median overall survival for these patients was not yet reached.

Another combination that merits further evaluation in the adjuvant setting, especially for patients with CSs, is the paclitaxel and carboplatin doublet. In a retrospective analysis, 49 women with completely resected stage I-IV CSs received adjuvantly either platinum-based chemotherapy (primarily paclitaxel and carboplatin) with or without radiation therapy or radiation therapy alone. Three-year PFS for chemotherapy group (with or without radiation therapy) was 35% versus 9% for radiation therapy alone, while the corresponding 3-year OS rates were 66% and 34%, respectively (HR = 2.02, p = 0.146) [36]. Furthermore, in a retrospective analysis of 26 patients with ovarian CSs, there was a trend for increased median survival for those who were treated with the paclitaxel and carboplatin combination (p = 0.066) [37].

More recently, trabectedin, an agent that binds to the DNA minor groove and has an original mechanism of action involving proteins of the nucleotide excision repair machinery has shown promising activity in patients with advanced STSs. Several trabectedin-based combinations have been tested in phase I trials, including combination with cisplatin, paclitaxel, docetaxel and pegylated doxorubicin [103]. Based on biological and/or histological features, trabectedin will be possibly utilized in the adjuvant setting in STSs, including uterine sarcomas.

The main studies conducted on adjuvant setting of early stage uterine sarcomas are presented in Table 1[4,6,21,33,35,43,44,94-102,104-106].

**Table 1:** Summary of studies on adjuvant chemotherapy in early-stage uterine sarcomas.

**Study**	**No of patients**	**Radiation**	**Regimens**	**Recurrence (%) at 5 years**	**5-year survival (%)**
Buchsbaum *et al*, 1979 [94]	8	NR	VAC	37	63
Omura *et al*, 1985 [100]	64	No	DOX	39	53
van Nagel *et al*, 1986 [95]	7	No	VAC	29	86
Berchuck *et al*, 1988 [6]	12 LMS	Yes	Various	83	42
Piver *et al*, 1988 [105]	11	No	CyVADIC	18	89
Hempling *et al*, 1995 [96]	20	No	CyVADIC	35	68
Resnik *et al*, 1995 [101]	23 CS	No	Etoposide + cisplatin + DOX	22	92 (at 2 years)
Gadducci et al, 1996 [43]	11 LMS	2 patients	Various	39	NR
Sartori et al, 1997 [106]	5 CS	No	NR	40	NR
Chauveinc et al, 1999 [21]	24	Yes	Various	NR	NR
Kushner et al, 2000 [98]	10	No	IFO	50	67 (at 3 years)
Manolitsas et al, 2001 [102]	21 CS	Yes	various	9.5	95
Giuntoli et al, 2003 [44]	34 LMS	2 patients	Various	NR	NR
Pautier et al, 2004 [99]	16	Yes	API	28	100
Odunsi et al, 2004 [97]	24	No	CyVADIC	33	75
Sutton et al, 2005 [35]	65 CS	No	IFO + Cisplatin	35	62
Ti et al, 2006 [104]	51	Yes	various	59	67
Papadimitriou et al, 2007 [4]	31	12 patients	Anthracycline-based	39	54
Wolfson et al, 2007 [33]	101 CS	No	CIM	52	46

## Conclusion

Uterine sarcomas represent a heterogeneous group of rare tumours that usually have an aggressive clinical behaviour and a poor prognosis. Newer classifications will help to distinguish the different types as we now recognise that carcinosarcomas are most likely to be of epithelial origin. The rarity of uterine sarcomas and their aggressive clinical course has resulted in a relatively limited amount of literature. Total abdominal hysterectomy and bilateral salpingo-oophorectomy remains the standard surgical care. Lymph node dissection is indicated for carcinosarcomas given their high incidence of lymph node metastases. Some recent data on small numbers of patients with low-grade endometrial stromal sarcomas appear to show an incidence of nodal involvement higher than previously expected, thus suggesting a role for lymphadenectomy. Adjuvant pelvic radiotherapy appears to improve local control without any significant impact on overall survival. Regarding adjuvant chemotherapy, there is little evidence in the literature supporting its use except for carcinosarcomas. However, more trials are needed to address these issues, especially, their sequential application. Based on some recent, albeit limited data, combinations such as paclitaxel and carboplatin in carcinosarcomas, as well as gemcitabine and docetaxel in leiomyosarcomas merit further evaluation in prospective trials of adjuvant treatment.

## Abbreviations

CIM: cisplatin-ifosfamide and mesna; CS: carcinosarcoma; CyVADIC: cyclophosphamide, vincristine, doxorubicin and dacarbazine; ER: estrogen receptor; ESS: endometrial stromal sarcoma; GOG: Gynecologic Oncology Group; LMS: leiomyosarcoma; PR: progesterone receptor; SEER: surveillance, epidemiology, and end results program; STS: soft tissue sarcoma; UUS: undifferentiated uterine sarcoma; VAC: vincristine, dactinomycin and cyclophosphamide; WAI: whole abdominal irradiation.

## Competing interests

The authors declare that they have no competing interests.

## Authors' contributions

FZ was involved in the writing of the manuscript (chemotherapy section), review of the literature, revised the manuscript. AMD revised the manuscript critically for important intellectual content. SF was involved in the writing of the manuscript (surgical treatment section), review of the literature. VK was involved in the writing of the manuscript (radiation therapy section), review of the literature. CAP was involved in conception of the idea, revised the manuscript critically for important intellectual content, final approval of the version to be published.
